# Multimorbidity and Its Effect on Self-Reported Adverse Effects Following COVID-19 Vaccination

**DOI:** 10.7759/cureus.33139

**Published:** 2022-12-30

**Authors:** Muhammad Riyyan, Sawaira Sajid, Sonika Hotwani, Hassan A Chania, Muhammad Shahzeb Shaikh, Yasir Sadiq, Hafiz A Sarwar, Aimen Azeem, Sikander M Memon, Shariq Abid

**Affiliations:** 1 Medical Research Center, Liaquat University of Medical and Health Sciences, Jamshoro, PAK; 2 General Practice and Public Health, Trauma Centre Hyderabad, Hyderabad, PAK; 3 Medicine and Surgery, Islam Medical College, Sialkot, PAK

**Keywords:** covid-19 vaccines, covid-19 vaccine side effects, sars-cov-2 vaccine reactogenicity, comorbidities and covid-19, multimorbidity

## Abstract

Introduction

The coronavirus disease 2019 (COVID-19) vaccination has been suggested for those with comorbidities, although there are concerns regarding the vaccine's safety. This study aimed to compare the severity and incidence of post-vaccination side effects in people with and without comorbidities. Another aim of this study was also to look for the effect of multimorbidity on adverse events.

Methods

This observational study was conducted from November 2021 to February 2022. Data were collected from all over Pakistan using a self-administered online questionnaire that inquired about the subject’s demographic, clinical, and COVID-19 vaccination profiles. Data analysis was done by using SPSS software version 22.0. (Chicago, IL, IBM Corp.).

Results

A total of 421 participants were included in the study, and 31.4% of individuals had underlying comorbidity. The overall mean age was 33 years (range: 13-85 years). This study included recipients of all major types of COVID-19 vaccines being used in Pakistan. Only 67.4% of the subjects had only underlying comorbidity, and hypertension was the most common one out of all comorbidities. Participants with comorbidities were not at a greater risk to produce vaccine-related adverse events when compared to those with no comorbidities. Comorbidity was also found to be statistically non-significant to the severity of the side effects. Only one subject with comorbidity produced a side effect and required hospitalization. Multimorbidity was not associated with a greater incidence of side effects. Multimorbidity was not significantly linked with the severity of the adverse effects, except muscle pain (p<0.05) and breathlessness (p<0.05).

Conclusion

It can be concluded that comorbidities do not affect the COVID-19 vaccine's reactogenicity but studies on an extensive scale should be conducted regarding individuals with multiple pre-existing comorbidities.

## Introduction

The new coronavirus disease 2019 (COVID-19), an infectious disease brought on by severe acute respiratory syndrome coronavirus 2 (SARS-CoV-2) has proven to be the biggest problem for public health services globally and has resulted in the death of over six million people [1]. Safe and effective SARS-CoV-2 vaccines with quick vaccination coverage are required to reach the herd immunity state and control this pandemic threat. Pakistan began its immunization campaign in February 2021, immunizing elderly people aged 70 years and older as the second category after frontline healthcare professionals [2].

​People with underlying comorbidities were more negatively affected by the COVID-19 infection. The risk of death and the severity of symptoms associated with COVID-19 are both exacerbated by comorbidities, and this claim has been supported by previously published studies. It has been reported earlier by Guan et al., in China that the majority of COVID-19-related deaths occurred in patients with any comorbidity [3]. The presence of more than one chronic health condition is called “multimorbidity.” Many studies provide evidence that the presence of multimorbidities increases the risk of hospitalization [4]. A study conducted in the setting of a Scotland hospital found that poor COVID-19 prognosis or worst clinical outcomes were observed in individuals with multimorbidities [5].

To prevent severe COVID-19 infection and reduce COVID-19-related hospitalizations, COVID-19 vaccines are crucial for people with chronic medical conditions [6]. Therefore, patients with serious comorbidities and immunocompromised status have been considered a too-priority group for COVID-19 primary and booster vaccination [7].

Despite increased uptake of COVID-19 vaccines worldwide, there’s still a remarkable proportion of vaccine hesitancy, including in this group of the population. World Health Organization (WHO) Strategic Advisory Group of Experts (SAGE) on immunization has described vaccine hesitancy as a “delay in acceptance or refusal of vaccination despite the availability of vaccination services” [8]. An internet-based survey assessed vaccine hesitancy by including patients suffering from cancer, diseases linked with autoimmunity, and chronic lung diseases and found that “newness of vaccine,” “safety of vaccine,” and “general distrust of the development process of vaccines” were the most common concerns [9]. Moreover, the fast production of vaccines and uncertainty about vaccines’ genetic composition further amplify these concerns [10]. Another common concern among this population is the vaccine's effect on underlying conditions and interactions with treatment [11,12]. Additionally, due to the paucity of data, there are increased concerns regarding vaccine safety in people with multimorbidities [13].

Thus, to resolve the conflicted attitudes toward COVID-19 vaccines among individuals with pre-existing medical risk conditions, this cross-sectional study was conducted to provide real-world data regarding the severity of the COVID-19 vaccine's side effects in this medically vulnerable population. The main objective of this study, therefore, is to compare and evaluate the severity and incidence of COVID-19 vaccine-related side effects in people with and without certain pre-existing medical conditions in Pakistan. The secondary objective is to compare the severity and incidence of vaccine-related adverse events in subjects with one comorbidity and those with more than one comorbidity.

## Materials and methods

Study design and participants 

We conducted a comparative cross-sectional study in Pakistan between November 2021 and February 2022. The study was approved by the Research Ethics Committee of Liaquat University of Medical and Health Sciences, REC# LUMHS/REC/159. The data collection via online survey started in December and was completed by January 2022. Out of 472 participants who filled out the online survey, only 421 met the inclusion criteria. Participants who were Pakistani nationals belonging to different provinces constituted the study population.

Inclusion and exclusion criteria 

Pakistani nationals of age 12 years or above who inoculated at least once for COVID-19 vaccine, approved by Pakistani authorities, met the study's inclusion criteria. Participants who were not immunized were subject to exclusion standards.

Sample size and data collection

With the use of the OpenEpi sample size calculator, a sample size of 421 was established while maintaining a level of significance, a 95% confidence interval, a 3.5% error, and the anticipated prevalence of fever, which the Centers for Disease Control and Prevention's research found to be 15.8% [14]. A non-probability sampling technique was employed for this investigation.

Data were collected using different online networking platforms and business contacts from all around Pakistan by using a standardized self-administered online questionnaire (SAQ), which was created on Google Forms. The questionnaire consisted of four main sections, the first section included consent and sociodemographic information, including age, gender, and place of residence; the second section included questions regarding the clinical profile of participants, including comorbidities (hypertension, diabetes mellitus, cardiac diseases, allergies, respiratory diseases, liver diseases, thyroid disorder, neurological disorder, immunosuppression, cancer, and transplantation history), and smoking history; the third section had a question regarding COVID-19 profile, including vaccination status (single or double dose) and type of vaccine (mRNA based including, Pfizer-BioNTech {BNT162b2} and Moderna {CX-024414}; inactivated virus-based including, Sinopharm {BBIBP-CorV} and Sino {CoronaVac}; viral-vector based including, Oxford-AstraZeneca {AZD1222}, CanSino Bio {Ad 5-nCoV}, and Sputnik V); and the fourth section had a question regarding the severity of post-vaccination side effects of COVID-19 vaccines. The scale of the severity for adverse effects following receipt of the COVID-19 vaccination was as follows: no side effect, no effect observed; mild, no treatment was required; moderate, treatment was required or advice from a healthcare professional in the outpatient department or outside of the hospital; and severe, the patient was hospitalized for care.

Statistical analysis

Statistical Package for the Social Sciences (SPSS) software version 22.0 (Chicago, IL, IBM Corp.) was used for the analysis of data. Qualitative data like demographic factors (gender, age, and residence), health status (smoking status and comorbidities), and COVID-19 vaccination status (number of doses and type of vaccines) were analyzed using descriptive statistics and were represented in terms of frequencies (n) and percentages (%). The mean (μ) is used to describe continuous variables like the participants' ages. Using the chi-squared test (χ2), it was possible to evaluate if there was a statistically significant relationship between the severity of the COVID-19 vaccine's post-vaccination side effects and comorbid conditions. To assess the incidence of COVID-19 post-vaccination adverse effects in people with single or multiple comorbidities, binary logistic regression was performed. All inferential analyses were performed with a 95% confidence interval (CI) and a significance threshold of 0.05.

## Results

From September to November 2021, 471 people answered the poll, but only 421 of them satisfied the standards for inclusion. This nationwide study where the subjects were selected from different provinces and the federal region includes 189 participants from Sindh province, 60 from Punjab province, 52 from Khyber Pakhtoun Khuwah (KPK) province, 45 from Balochistan province, 34 from Gilgit Baltistan region and 41 from the capital Islamabad. The demographic distribution of the selected individuals is shown in Table 1, which also reports that 369 people out of 421 were non-smokers as compared to 52 smokers.

**Table 1:** Demographic variables of participants stratified according to presence or absence of comorbidities. Here, "n" denotes the number of participants and "%" denotes the percentage of participants. KPK: Khyber Pakhtoun Khuwah

Variables		Comorbidities, n (%)	No comorbidities, n (%)
Age groups of subjects	≤33	42 (31.8%)	176 (60.9%)
	>33	90 (68.2%)	113 (39.1%)
Gender of subject	Male	85 (64.4%)	181 (62.6%)
	Female	47 (35.6%)	108 (37.4%)
Type of vaccine	Inactivated	48 (36.4%)	78 (27%)
	mRNA	45 (34.1%)	110 (38.1%)
	Viral vector	39 (29.5%)	101 (34.9%)
Smoking history of subjects	Yes	20 (15.2%)	32 (11.1%)
	No	112 (84.8%)	257 (88.9%)
Residency of subjects	Sindh	62 (47%)	127 (43.9%)
	Punjab	20 (15.2%)	40 (13.8%)
	KPK	18 (13.6%)	34 (11.8%)
	Islamabad	7 (5.3%)	34 (11.8%)
	Balochistan	13 (9.8%)	32 (11.1%)
	Gilgit Baltistan	12 (9.1%)	22 (7.6%)
Vaccination status of subjects	First	37 (28%)	82 (28.4%)
	Second	95 (72%)	207 (71.6%)

By the time of completing the online questionnaire, underlying comorbidity was present in 132 participants, of whom 37 had received the first dose and 95 received the second dose of the vaccine. Out of 289 individuals who had no underlying comorbidity, 82 had their first dose, and 207 had received a second. Regarding vaccine type, in participants without underlying comorbidity, 78 had the inactive vaccine, 110 had the mRNA vaccine, and 101 received the viral vector vaccine, whereas, in participants with comorbidity, 48 participants received the inactive vaccine, 45 participants received the mRNA vaccine, and 39 participants received viral vector vaccine.

When compared, 43 subjects had multiple comorbidities and 89 had only one. Hypertension was the most common comorbidity among participants, accounting for 36.4%, followed by diabetes mellitus with 32.6%, whereas, 24.2% presented with allergic responses, 16.7% were immune-suppressed, 9.8% were having cardiac diseases, 7.6% were effected with neurological diseases, 6.8% individuals were having pulmonary diseases, and 5.3% were of liver diseases, and the least frequently mentioned were with malignancies or cancer which accounts for only 3% of the individuals, with none of the selected individuals were having any transplantation history.

As demonstrated in Table 2, participants with and without comorbidities were compared in terms of the incidence of self-reported vaccination adverse effects. Although the results of the logistic regression showed statistical non-significances, participants with comorbidities had a higher incidence of self-reported cough, followed by flu-like illness, allergic reactions, diarrhea, joint pain, fever, and injection site swelling, while a lower incidence of the remaining self-reported side effects.

**Table 2:** Severity and Incidence of COVID-19 vaccine-related side effects among participants with and without comorbidities. COVID-19: coronavirus disease 2019

Side effect	Comorbidities				No comorbidities				p-Value	Incidence of side effect: odd ratio (95% CI)
	No side effect, n (%)	Mild, n (%)	Moderate, n (%)	Severe, n (%)	No side effect, n (%)	Mild, n (%)	Moderate, n (%)	Severe, n (%)		
Fever	87 (65.9%)	29 (15.9%)	24 (18.2%)	0 (0%)	194 (67.1%)	43 (14.9%)	52 (18%)	0 (0%)	0.958	1.110 (0.719-1.713)
Chills	130 (98.5%)	1 (0.8%)	1 (0.8%)	0 (0%)	277 (95.8%)	10 (3.5%)	2 (0.7%)	0 (0%)	0.272	0.588 (0.161-2.143)
Cough	127 (96.2%)	4 (3%)	1 (0.8%)	0 (0%)	284 (98.3%)	4 (1.4%)	1 (0.3%)	0 (0%)	0.437	2.236 (0.636-7.861)
Headache	119 (90.2%)	7 (5.3%)	6 (4.5%)	0 (0%)	255 (88.2%)	16 (5.5%)	17 (5.9%)	1 (0.3%)	0.850	0.920 (0.475-1.784)
Flu-like illness	123 (93.2%)	5 (3.8%)	4 (3%)	0 (0%)	276 (95.5%)	8 (2.8%)	5 (1.7%)	0 (0%)	0.585	1.892 (0.796-4.497)
Hypotension	130 (98.5%)	2 (1.5%)	0 (0%)	0 (0%)	278 (96.2%)	10 (3.5%)	1 (0.3%)	0 (0%)	0.426	0.389 (0.085-1.779)
Fatigue	103 (78%)	20 (15.2%)	9 (6.8%)	0 (0%)	221 (76.5%)	54 (18.7%)	14 (4.8%)	0 (0%)	0.517	0.975 (0.597-1.591)
Muscle pain	118 (89.4%)	11 (8.3%)	3 (2.3%)	0 (0%)	252 (87.2%)	26 (9%)	11 (3.8%)	0 (0%)	0.692	0.901 (0.475-1.710)
Joint pain	123 (93.2%)	7 (5.3%)	2 (1.5%)	0 (0%)	272 (94.1%)	13 (4.5%)	4 (1.4%)	0 (0%)	0.931	1.171 (0.508-2.700)
Breathlessness	130 (98.5%)	2 (1.5%)	0 (0%)	0 (0%)	284 (98.3%)	4 (1.4%)	1 (0.3%)	0 (0%)	0.791	0.874 (0.167-4.563)
Nausea and vomiting	129 (97.7%)	1 (0.8%)	2 (1.5%)	0 (0%)	281 (97.2%)	5 (1.7%)	3 (1%)	0 (0%)	0.678	0.817 (0.213-3.130)
Diarrhea	130 (98.5%)	2 (1.5%)	0 (0%)	0 (0%)	286 (99%)	2 (0.7%)	1 (0.3%)	0 (0%)	0.576	1.467 (0.242-8.883)
Injection site pain	87 (65.9%)	28 (21.2%)	17 (12.9%)	0 (0%)	183 (63.3%)	70 (24.2%)	36 ( 12.5%)	0 (0%)	0.795	0.937 (0.609-1.442)
Injection site swelling	119 (90.2%)	10 (7.6%)	3 (2.3%)	0 (0%)	258 (89.3%)	26 (9%)	5 (1.7%)	0 (0%)	0.834	1.024 (0.524-2.003)
Allergic reactions	128 (97%)	1 (0.8%)	2 (1.5%)	1 (0.8%)	284 (98.3%)	4 (1.4%)	1 (0.3%)	0 (0%)	0.236	1.775 (0.469-6.720)
Tachycardia	130 (98.5%)	1 (0.8%)	1 (0.8%)	0 (0%)	284 (98.3%)	5 (1.7%)	0 (0%)	0 (0%)	0.248	0.874 (0.167-4.563)
Menstrual cycle disturbances	129 (97.7%)	3 (2.3%)	0 (0%)	0 (0%)	280 (96.9%)	8 (2.8%)	1 (0.3%)	0 (0%)	0.760	0.724 (0.193-2.717)

Moreover, individuals with and without comorbidities were compared for COVID-19 vaccination adverse reactions in terms of severity, the chi-square test showed that comorbidity was statistically independent of the severity of side effects (p>0.05). The percentage of subjects who reported COVID-19 vaccine adverse events was similar in the group with and without comorbidities. Furthermore, adverse effects that necessitated hospitalization, categorized as severe, were rarely reported in either group, except for one individual without comorbidity who needed hospitalization for a headache, in addition to the one with underlying comorbidity having an allergic response.

As demonstrated in Table 3, individuals with only one comorbidity and those with multimorbidity were compared in terms of the severity and incidence of self-reported vaccination adverse effects. According to the chi-square test, participants with multimorbidity were associated to experience greater severity of muscle pain, p=0.045, and breathlessness, p=0.04, while no association was among other observed COVID-19 vaccine-related side effects (p>0.05). No statistical significance was demonstrated because the lower limit of 95% could not be determined, however, among people who had multimorbidity, there was a lower incidence of flu-like illness, injection site pain, fever, fatigue, joint pain, headache, and muscle pain. Data also show that there was a higher incidence of self-reported side effects of allergic reactions, followed by nausea and vomiting, menstrual cycle disturbance, diarrhea, hypotension, coughing, and chills. Breathlessness and tachycardia were not reported by any individuals with one comorbidity, hence tests to determine the incidence could not be used.

**Table 3:** Severity and incidence of COVID-19 vaccine-related side effects among participants with one or more than one comorbidity. COVID-19: coronavirus disease 2019

Side effect	Greater than one comorbidity				One comorbidity				p-Value	Incidence of side effect: odd ratio (95% CI)
	No side effect, n (%)	Mild, n (%)	Moderate, n (%)	Severe, n (%)	No side effect, n (%)	Mild, n (%)	Moderate, n (%)	Severe, n (%)		
Fever	29 (67.4%)	6 (14%)	8 (18.6%)	0 (0%)	57 (64%)	15 (16.9%)	17 (19.1%)	0 (0%)	0.901	0.860 (0.398-1.859)
Chills	42 (97.7%)	1 (2.3%)	0 (0%)	0 (0%)	87 (97.8%)	1 (1.1%)	1 (1.1%)	0 (0%)	0.684	1.036 (0.091-11.747)
Cough	41 (95.3%)	2 (4.7%)	0 (0%)	0 (0%)	86 (96.6%)	2 (2.2%)	1 (1.1%)	0 (0%)	0.594	1.398 (0.225-8.695)
Headache	40 (93%)	2 (4.7%)	1 (2.3%)	0 (0%)	78 (87.6%)	5 (5.6%)	6 (6.7%)	0 (0%)	0.546	0.532 (0.140-2.015)
Flu-like illness	40 (93%)	2 (4.7%)	1 (2.3%)	0 (0%)	82 (92.1%)	4 (4.5%)	3 (3.4%)	0 (0%)	0.947	0.879 (0.216-3.578)
Hypotension	42 (97.7%)	1 (2.3%)	0 (0%)	0 (0%)	88 (98.9%)	1 (1.1%)	0 (0%)	0 (0%)	0.596	2.095 (0.128-34.320)
Fatigue	35 (81.4%)	4 (9.3%)	4 (9.3%)	0 (0%)	67 (75.3%)	17 (19.1%)	5 (5.6%)	0 (0%)	0.291	0.696 (0.281-1.723)
Muscle pain	41 (95.3%)	0 (0%)	2 (4.7%)	0 (0%)	76 (85.4%)	11 (12.4%)	2 (2.2%)	0 (0%)	0.045	0.285 (0.061-1.325)
Joint pain	41 (95.3%)	2 (4.7%)	0 (0%)	0 (0%)	82 (92.1%)	5 (5.6%)	2 (2.2%)	0 (0%)	0.592	0.571 (0.114-2.875)
Breathlessness	41 (95.3%)	2 (4.7%)	0 (0%)	0 (0%)	89 (100%)	0 (0%)	0 (0%)	0 (0%)	0.04	-
Nausea and vomiting	41 (95.3%)	0 (0%)	2 (4.7%)	0 (0%)	88 (98.9%)	1 (1.1%)	0 (0%)	0 (0%)	0.097	4.293 (0.378-48.706)
Diarrhea	42 (97.7%)	1 (2.3%)	0 (0%)	0 (0%)	88 (98.9%)	1 (1.1%)	0 (0%)	0 (0%)	0.596	2.095 (0.128-34.320)
Injection site pain	29 (67.4%)	10 (23.3%)	4 (9.3%)	0 (0%)	57 (64%)	18 (20.2%)	14 (15.7%)	0 (0%)	0.59	0.860 (0.398-1.859)
Injection site swelling	38 (88.4%)	3 (7%)	2 (4.7%)	0 (0%)	80 (89.9%)	8 (9%)	1 (1.1%)	0 (0%)	0.42	1.170 (0.367-3.729)
Allergic reactions	40 (93%)	1 (2.3%)	1 (2.3%)	1 (2.3%)	88 (98.9%)	0 (0%)	1 (1.1%)	0 (0%)	0.211	6.600 (0.666-65.424)
Tachycardia	41 (95.3%)	1 (2.3%)	1 (2.3%)	0 (0%)	89 (100%)	0 (0%)	0 (0%)	0 (0%)	0.122	-
Menstrual cycle disturbances	41 (95.3%)	2 (4.7%)	0 (0%)	0 (0%)	88 (98.9%)	1 (1.1%)	0 (0%)	0 (0%)	0.202	4.293 (0.378-48.706)

## Discussion

We found that pre-existing comorbidities were not linked with the incidence and severity of the side effects produced by COVID-19 vaccines. Individuals who had comorbidities were not at a greater risk to develop post-vaccination complications and neither they reported more severe side effects when compared to participants without comorbidities. Similarly Beg et al. reported in a survey from Pakistan that participants who had comorbidities did not experience more vaccine-related adverse effects, compared to the participants who had no history of such diseases [15]. Approximately 70% of the participants in our cohort who had pre-existing chronic conditions were older than the mean age calculated in our study. The survey conducted by Beg et al. also included participants mainly of older age groups.

On the contrary, multiple studies around the globe illustrated that people who had pre-existing comorbidities produced a greater number of post-vaccination side effects [16,17]. Mainly these studies included a greater number of young participants. Like one of these, a survey conducted in Pakistan by Abbas et al. showed that comorbidities were significantly linked with incidence of vaccine side effects, but interestingly the upper age limit of the participants in this study was only 55 years and majority of them consisted of young individuals [18]. Probably the age of the individuals might be one of the reasons for these contrasting results between different studies conducted around the world. According to past literature and one meta-analysis conducted, authors highlighted age stratification according to vaccine reactogenicity and one out of three stratified groups consisted of individuals older than 55 years [19,20]. Research conducted by Abbas et al. did not report the outcomes from any single individual from this stratified group. The above-mentioned explanation should be considered since multiple international studies have uniformly concluded that young age is significantly linked with greater reactogenicity of the COVID-19 vaccines, and therefore role of the age, its correlation with comorbidities, and its effect on the side effects profile among this group of population must be thoroughly investigated [18,21,22].

Secondarily, we found that multimorbidity in a participant is not associated with a greater incidence of post-vaccination side effects compared to single comorbidity. However, participants who had more than one comorbidity did not suffer from more severe side effects compared to single comorbidity except for two side effects, which were muscle pain and breathlessness, but these two were majorly moderate in severity and none of the participants required hospital care for these side effects. According to our literature search, not many studies have compared the COVID-19 vaccine reactogenicity according to the number of comorbidities. However, a study published in nature communications found no evidence for multimorbidity posing greater risk of COVID-19 vaccine side effects but this study only included two vaccines used in Hong Kong [23]. Also, this study did not consider and compared the severity of the adverse events among people with multimorbidity and single comorbidity.

The findings of our study display a very positive trend that very few participants reported severe adverse effects and even those who did had no association with pre-existing comorbidities. These findings have a great implication for population living especially in Pakistan and in other low-/middle-income countries where prevalence of comorbidities is very high. These findings would not just reduce the hesitancy for the COVID-19 vaccines, rather they would have an impact on all kinds of vaccines rollout among people suffering from chronic diseases. One systematic review highlighted that approximately one-third of the global public is suffering from multimorbidity [24]. Outcomes of our study would help out this major chunk of population, by decreasing the possible fears and increasing their trust in global vaccination programs including COVID-19. As discussed earlier, comorbidity is significantly linked with greater mortality among individuals infected with the virus, by boosting confidence towards vaccine safety and increasing vaccine outreach, this study would help in lowering hospitalization and death rates in the most vulnerable group of COVID-19 patients.

There are various strengths of this study that need to be considered. Firstly, this study is the first one to be conducted in Pakistan that has looked for association between multimorbidity and post-COVID-19 vaccination side effects. This study stands out as it has analyzed not only incidence of side effects but also compared the severity of side effects between participants who had single or multiple comorbidities and participants who had none. This study included individuals who had been vaccinated from all types of COVID-19 vaccines approved by the government of Pakistan and WHO across the globe, and there were approximately equal responses from each type of vaccine which consequently eliminated vaccine bias.

Primarily our study relies on self-reported and self-assessed responses and some participants may not have appropriately reported all the data, therefore, potential response bias is one of the limitations. The study lacks detail regarding comorbidities status and the duration of these chronic illnesses existed for. The study did not analyze the correlation between the number of doses, onset of the comorbidity, and incidence of side effects. At the time of data collection, booster doses were not approved by the government of Pakistan, hence the effect of booster doses was not considered among the participants.

However, in the future, multiple studies should be conducted and those should include a somewhat uniform number of individuals from each group stratified according to age, so that correlation between age and its effect on reactogenicity among subjects with history of comorbidities can be identified. Moreover, future researchers should also consider multimorbidity as a rising global phenomenon, and many projects should be carried out in order to know more about the connection between the number of pre-existing comorbidities and its potential effect on side effects of various vaccines.

## Conclusions

It can be concluded that a history of comorbidities is not significantly linked with the greater incidence and severity of post-COVID-19 vaccination adverse effects. Individuals with multimorbidity are not at a greater risk to develop vaccine-related side effects, and multimorbidity has no increased effect on the severity of adverse events except breathlessness and tachycardia but only one participant with pre-existing comorbidities reported any severe adverse event. This study would help to decrease vaccine hesitancy among this vulnerable group of the population, as it reports findings of those people too who are suffering from multiple chronic illnesses. However, more research should be conducted, assessing vaccine reactogenicity according to the number of comorbidities, through which acceptance of upcoming vaccination programs could be increased in this population of the world.
